# Hierarchical clustering of MS/MS spectra from the firefly metabolome identifies new lucibufagin compounds

**DOI:** 10.1038/s41598-020-63036-1

**Published:** 2020-04-08

**Authors:** Catherine Rawlinson, Darcy Jones, Suman Rakshit, Shiv Meka, Caroline S. Moffat, Paula Moolhuijzen

**Affiliations:** 1 Centre for Crop and Disease Management, School of Molecular and Life Sciences, Curtin University, Bentley, Western Australia Australia; 2 Statistics for the Australian Grains Industry-West, School of Molecular and Life Sciences, Curtin University, Bentley, Western Australia Australia; 3 Curtin Institute for Computation, Curtin University, Bentley, Western Australia Australia

**Keywords:** Mass spectrometry, Metabolomics, Software

## Abstract

Metabolite identification is the greatest challenge when analysing metabolomics data, as only a small proportion of metabolite reference standards exist. Clustering MS/MS spectra is a common method to identify similar compounds, however interrogation of underlying signature fragmentation patterns within clusters can be problematic. Previously published high-resolution LC-MS/MS data from the bioluminescent beetle (*Photinus pyralis*) provided an opportunity to mine new specialized metabolites in the lucibufagin class, compounds important for defense against predation. We aimed to 1) provide a workflow for hierarchically clustering MS/MS spectra for metabolomics data enabling users to cluster, visualise and easily interrogate the identification of underlying cluster ion profiles, and 2) use the workflow to identify key fragmentation patterns for lucibufagins in the hemolymph of *P. pyralis*. Features were aligned to their respective MS/MS spectra, then product ions were dynamically binned and resulting spectra were hierarchically clustered and grouped based on a cutoff distance threshold. Using the simplified visualization and the interrogation of cluster ion tables the number of lucibufagins was expanded from 17 to a total of 29.

## Introduction

Metabolomics is the scientific study of the low molecular weight compounds (metabolites) within an organism, cell or tissue, which reflect underlying biochemical activities and cellular processes. A major challenge of metabolomic analysis is the identification of these compounds. If the reference MS/MS spectrum for a metabolite is not publicly or commercially available, identification is unlikely. However, compounds of similar structure often have similar MS fragmentation pathways leading to mass spectral patterns specific to a chemical class.

As an example, triticones are a class of specialized metabolites produced by the necrotrophic fungal pathogen, *Pyrenophora tritici-repentis* (Ptr), which have recently been functionally characterised^1^. Since triticones were first purified in 1988, several have been purified and characterized via NMR analyses. However, it is only recently that their MS/MS spectra have been explored which enabled the putative identification of a total of 38 triticones in the LC-MS/MS profile of Ptr. It is important to understand the complement of structurally similar bioactive molecules produced by an organism as activity across a class of compounds may offer customized responses to biological stressors.

With the introduction of acquisition techniques collecting MS/MS spectra with no prior knowledge of sample composition, thousands of unique spectra can be generated from a single sample. With substantially higher MS/MS coverage of features, information about chemical structure can be leveraged from repeated mass spectral patterns, aiding in classification of unknown metabolites. However, thorough exploration of MS/MS data generated with these new techniques is often cumbersome and impractical. Even after statistical analyses has derived a list of analytes of interest, correlating their mass spectra to other analytes can be laborious.

Several tools have been developed to assist with MS/MS pattern recognition. Molecular networking-based visualization is becoming increasingly popular in metabolomics and is used by tools such as Global Natural Products Social Molecular Networking (GNPS)^2–4^. Whilst use of such tools is becoming more prevalent, GNPS is web-based requiring upload of data to a server and is limited in parameter customization of workflow and little in exportable, easy to interrogate results. ‘MetCirc” is an R based package^5^ offering clustering of MS/MS spectra and uses a Circos plot for visualization^6^. However, because MetCirc uses a defined number of identically sized “fixed” bins, instrumental variability and precision may lead to incorrectly binned ions causing false or incomplete conclusions^7–9^. This motivated us to create a workflow that was easy to use and to interrogate fragmentation profiles using dynamic binning, which allows instrument resolution and precision to inform binning, and hierarchically clustering of MS/MS spectra.

In this study, we demonstrate the effectiveness of hierarchically clustering MS/MS spectra for the discovery of underlying ion profiles to identify or classify unknown metabolites. A previously published dataset of firefly predator defense lucibufagin compounds^10^ was reanalysed using dynamic binning and hierarchical clustering, and results were compared to the gold standard web-based Feature Based Molecular Networking (FBMN) module of GNPS. We provide the techniques used as a simple but effective workflow, BioDendro (https://github.com/ccdmb/BioDendro), that users with minimal coding skills can easily use and customize to identify core fragmentation patterns.

## Material and Methods

### LC-MS/MS Firefly Metabolights data source

LC-MS/MS data was sourced from the MetaboLights repository, project ID MTBLS698 (https://www.ebi.ac.uk/metabolights/MTBLS698)^10^ for the analysis of luminescent and non-luminescent tissue of beetle species^10^. Data were collected on a Thermo Q-Exactive Orbitrap using data dependent acquisition (DDA) with polarity switching using a C18 column^10^. A single file generated from the hemolymph of an adult male *Photinus Pyralis* beetle was selected (Ppyr_hemolymph_extract.mzML). The positive ion mode analysis was previously carried out using MZmine (v2.30) with MS^2^ similarity search and published using the parameters described in section 4.6 of the supplementary information^10^. The positive ion data for Ppyr_hemolymph_extract.mzML was reanalyzed here with MZmine2 (v2.53) using the same settings. Parameters which have changed or added between versions were applied as was suitable for the data (Supplementary Table S1). To identify new lucibufagins, hierarchical clustering of MS/MS spectra with subsequent visualization and interrogation was applied using a newly created workflow application, BioDendro. Results from BioDendro were then compared to molecular networking of MS/MS spectra using FBMN^4^ module of GNPS^2^.

### Firefly LC-MS/MS analysis using BioDendro workflow

BioDendro released under an Apache 2.0 license is available for download at https://github.com/ccdmb/BioDendro. BioDendro requires the use of Python3 and is run locally through the provided Jupyter Notebook (https://jupyter.org/) to execute the programs workflow. Both applications can be downloaded and installed through the package management tool, Anaconda (https://www.anaconda.com/distribution/). Detailed instructions on download, installation and usage of BioDendro can be found in GitHub (https://github.com/ccdmb/BioDendro). A detailed explanation of parameters and recommended settings can be found in Supplementary Information SI1. Two Jupyter notebooks have been supplied; “quick-start-example.ipynb” which contains all the settings applied herein and “longer-workflow.ipynb” which provides a set by step execution.

BioDendro requires two input text files to function; a file containing all the features within a data set (.txt) and MS/MS spectra in MGF format (.mgf) (Supplementary Information SI1). A single MS/MS spectrum was aligned to a feature based on a mass (*m/z* 0.005) and retention time (6 secs) user defined tolerances, where multiple matches exist, the closest in retention time was associated using pandas core package^11^. Two optional steps can be applied at this stage; an absolute/relative filtering of ions and application of neutral loss formatting to spectra. An absolute filtering of ions (minimum intensity of 5000) and no neutral loss was performed. Prior to comparison of spectra, all masses were binned to allow appropriate comparison of spectra using variable bin sizes and the numpy core package^12^. All product ions were ordered by *m/z* and a new bin was created when the difference between 2 consecutive masses exceeded a user defined threshold (defined here as *m/z* 0.0005). This value should reflect instrument precision. Pairwise distances were then calculated between all binned spectra using the Bray-Curtis metric implemented in scipy (Jaccard distance is also available; see S2 for description of these metrics)^13^. The distance matrix then hierarchically clustered using complete-linkage clustering implemented in scipy^13^. A user specifiable distance threshold can be used to select clusters from the hierarchically clustered data. Lastly, data were visualized as a tree using plotly^14^ and the user defined distance threshold was set to 0.7. Resulting clusters were output as ion histograms using matplotlib^15^ and in tabular format that represented clustered features and the associated MS/MS spectra. See Supplementary Table S2 for a summary of analysis parameters.

### Firefly LC-MS/MS analysis using FBMN module of GNPS

Data were extracted from Ppyr_hemolymph_extract.mzML for analysis in the FBMN module of GNPS analysis platform^2,4,16^. Documentation for analysis using FBMN with MZmine2 can be found at https://ccms-ucsd.github.io/GNPSDocumentation/featurebasedmolecularnetworking-with-mzmine2/. Two text files are required for analysis with FBMN, a.txt file containing the sample features and the aligned MS/MS spectra in MGF. The files were generated using MZmine2 as per the parameters described by Fallon, *et al*.^10^. Molecular networking was carried out using BioDendro settings where applicable (Supplementary Table S2).

### Comparison of BioDendro and FBMN

Comparisions of BioDendro and FBMN used the same feature list generated by MZmine2 as per the analysis settings in Supplementary Table S2. BioDendro used an MGF file produced from the freeware ProteoWizard^17^, which exports all MS/MS spectra collected. For FBMN, an MGF of aligned MS/MS spectra were exported from MZmine2 as required.

Comparative analysis of clustering between BioDendro and FBMN was accomplished in a targeted manner by comparing the clustering of the putatively identified lucibufagin class of compounds. This involved a manual search using retention time and precursor mass to locate each feature within the respective pipelines.

Analyses using BioDendro and FBMN were carried out using a Windows 7 64-bit PC. The PC had an Intel i7 processor and 16GB of RAM.

### Metabolite classification and identification

Putative identification of lucibufagins by Fallon, *et al*.^10^ was by targeted search of masses in the LC-MS profile of known lucibufagin compounds and then expanded upon by MS^2^ similarity searching in MZmine2. The putatively identified lucibufagins and their respective MS/MS spectra was employed herein. METLIN^18^, MassBank^19^ and NIST14^20^ mass spectral databases were searched for reference spectra. Literature was also searched for mass spectral information pertaining to “lucibufagin MS/MS”.

The molecular formula for fragment ions were predicted using the ‘Elemental composition” function within the Qual Browser module of Thermo Xcalibur software. Details for prediction are outlined in Supplementary Information SI2. Fallon, *et al*.^10^ reported greatest instrumental error as +9.9 ppm for tryptophan (*m/z* 205.09) and therefore a ±10 ppm tolerance was used. Elemental predictions were limited to formula containing only C, H and O as all reported lucibufagins by Fallon, *et al*.^10^ contained only these elements.

CSI:FingerID^21^ within the SIRIUS 4.0.1 GUI^22^ was used to explore possible structures for fragmentation patterns of unknown lucibufagins.

## Results

The BioDendro workflow (Supplementary Figure S1) was applied to the positive ion mode acquisition of a single sample, Ppyr_hemolymph_extract.mzML, within the project dataset representing the hemolymph of an adult male beetle, *Photinus pyralis* spp. Examination of the raw data for Ppyr_hemolymph_extract.mzML, showed the acquisition of 2,501 MS/MS spectra, of which 1,251 were collected in positive ion mode. Deconvolution of the sample in MZmine2 (v2.53) extracted 29,677 features for positive ion mode when identified isotopes were excluded.

### Clustering the lucibufagins in P.pyralis hemolymph

To identify new lucibufagins (Supplementary Figure S2) in the hierarchically clustered MS/MS spectra we first focused on a comparison against the original analysis. Fallon, *et al*.^10^ putatively identified 17 lucibufagin compounds in the *P. pyralis* hemolymph using MZmine2 (v2.30) that varied by the degree of substitution of hydroxyl groups with acetyl and propyl groups. These lucibufagins were putatively identified by a combination of accurate mass, retention time and MS/MS spectra. Analysis with MS^2^ similarity search in MZmine2 (v2.30) aligned 9 of the 17 lucibufagins to an MS/MS spectrum and the remaining 8 were defined by precursor mass and retention time.

**Figure 1:**
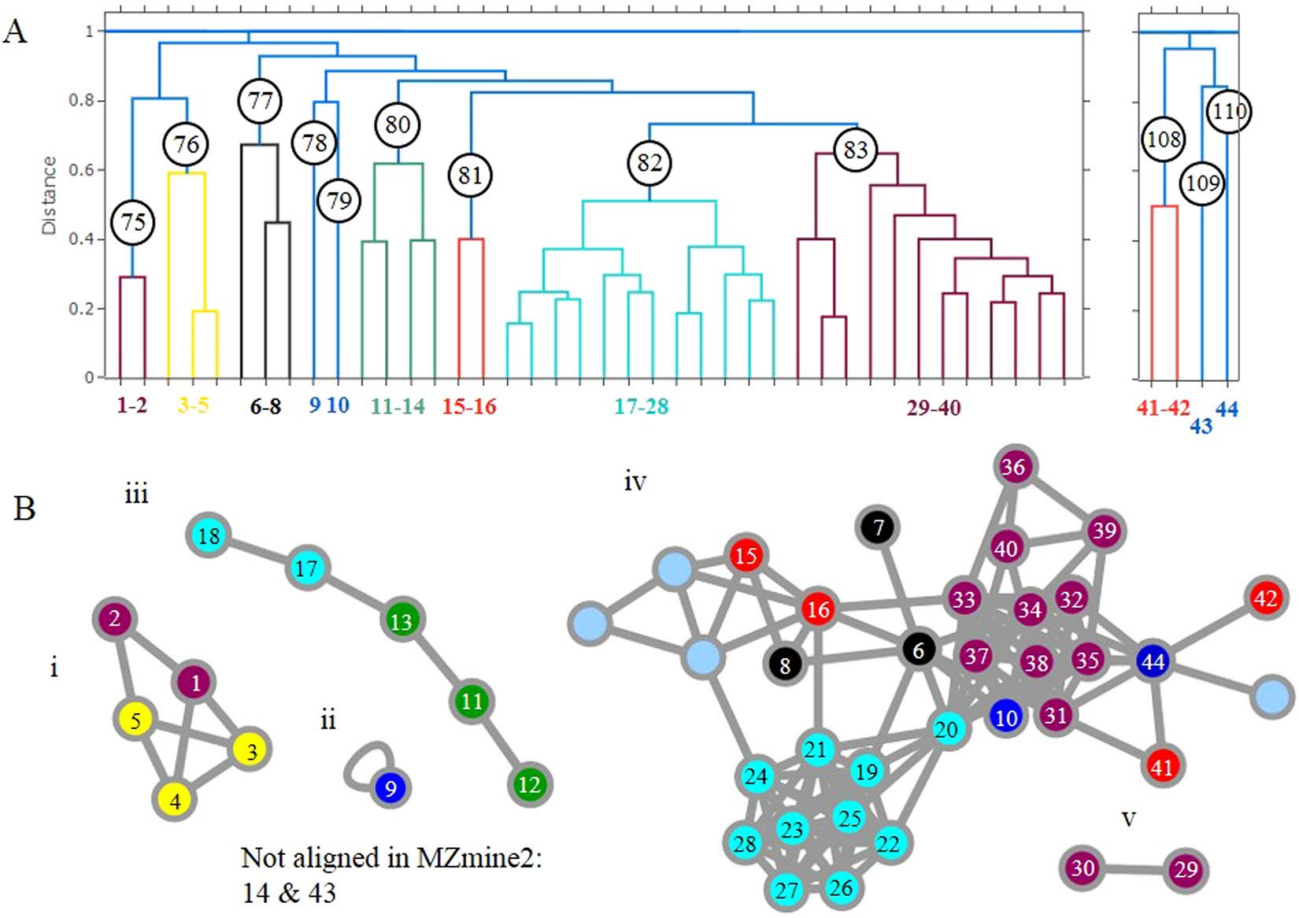
Clustering of Ppyr_hemolymph_extract.mzmL MS/MS spectra using (**a**) BioDendro complete linkage hierarchical clustering using a distance threshold of 0.7 and (**b**) FBMN molecular networking using a cosine score of 0.7. Features have been arbitrarily named from 1 to 44 to show the position in both tree and networks and represented in Table 1. The network nodes have been coloured to represent the same feature colour in the tree. Nodes without numbers are not clustered within the tree branches.

A targeted search for the putatively identified compounds from Fallon, *et al*.^10^ using BioDendro revealed that 15 of the 17 targets had been assigned an MS/MS spectra during alignment and had been placed into 4 clusters identified as clusters 82, 83, 108, and 110 (Fig. 1a). There was a total of 27 features within these 4 clusters, of which 11 of the 12 additional features represented adducts or aggregate ions that had been manually removed from the original analysis and a single new dipropylated isomer that was found beyond the retention time analyzed by Fallon, *et al*.^10^.

Clusters 82 and 83 represented the largest with 12 features each and contained 12 of the original lucibufagins identified by Fallon, *et al*.^10^. The remaining 2 clusters (108 and 110) both contain core lucibufagin isomers and a single monoacetylated isomer, clustered away from the main branch in cluster 108 and 110. Inspection of associated ion tables for cluster 82 and 83, showed that 4 ions were present in every feature of both clusters (m/z 105.0701, 121.0648, 147.0805, 185.0961) (Supplementary Dataset). In addition, there were 3 ions unique to and present in every feature of cluster 82 (m/z 135.0443, 205.0863 and 413.1965) and 2 ions for cluster 83 (m/z 151.0392 and 265.1592) (Table 1). Only a single molecular formula for each fragment mass was predicted using the parameters detailed in Supplementary Information S2. These ions were considered diagnostic of a lucbibufagin-like structure. METLIN^18^, MassBank^19^ and NIST 14^20^ spectral databases were searched for “lucibufagin” in an attempt to corroborate putative identification of these compounds however, for all 3 searches, zero hits were returned. A search of PubChem^23^ and ChemSpider^24^ had entries for lucibufagin C and several compounds with molecular similarity but no MS/MS spectral data was found. A literature search for “lucibufagins MS/MS” found several papers containing mass spectral information of several purified and characterized lucibufagins^25–27^. A search for the highly represented ions in these publications showed the presence of these ions.

**Table 1:** Ions that show 100% representation in features of clusters 82 and 83 with their predicted molecular formula and ppm error compared to the average fragment ion mass.

Fragment ion (m/z average_m/z min_m/z max)	% present		Predicted molecular formula	ppm error	Incidence in 44 features (%)
	cluster 82	cluster 83			
135.0443_135.0434_135.0448	100	0	C8H7O2	1.8	16 (36%)
205.0863_205.0840_205.0872	100	0	C12H13O3	1.8	18 (41%)
413.1965_413.1950_413.1970	100	0	C24H29O6	1.5	15 (34%)
105.0701_105.0698_105.0707	100	100	C8H9	2.1	36 (82%)
121.0648_121.0644_121.0653	100	100	C8H9O	0.1	41 (93%)
147.0805_147.0756_147.0812	100	100	C10H11O	1.1	35 (80%)
185.0961_185.0924_185.0975	100	100	C13H13O	−1.0	27 (61%)
151.0392_151.0388_151.0396	0	100	C8H7O3	1.5	14 (32%)
265.1592_265.1540_265.1670	0	100	C19H21O	1.9	17 (39%)

The incidence of these ions from cluster 82 and 83 was scrutinized in surrounding clusters. A total of 12 clusters (75–83, 108–110), comprising 44 features (inclusive of the original 27) were shown to have a complement of these ions (Tables 1 and S3). The hierarchically clustered tree revealed clusters 75–83 belonged to the same cluster when the tree distance threshold was set at 0.97 (Fig. 1a).

Retention time was used to identify features with likely multiple adducts and confirmed through comparison of the calculated mass for the proposed adducts. The 44 features represented 29 unique compounds, including 15 from the original analysis and 14 from the re-analysis using BioDendro (Table 2).

**Table 2:** Features of the lucibufagin clusters 75–83 and 108–110. The feature list ID was aligned to the putative ID in Fallon, *et al*.^10^. Additional features were assigned a putative identity based on comparison to the original analysis and a calculated molecular formula.

Feature^a^	Cluster #	Feature List ID	Fallon *et al*. (2018) putative identification	Putative classification	Adduct^b^	Aligned MS/MS in MZmine2^c^	FBMN cluster ID (as per Fig. 1)^d^	Molecular Formula^e^
1	75	Ppyr_hemolymph_extract_436.269439697265_13.538295		unknown 1	M + H		i	C24H37NO6
2	75	Ppyr_hemolymph_extract_453.296081542968_13.538295		adduct of unknown 1	M + NH4		i	C24H37NO6
3	76	Ppyr_hemolymph_extract_434.25390625_13.471111		unknown 2	M + H		i	C24H35NO6
4	76	Ppyr_hemolymph_extract_452.264434814453_8.0686129		unknown 3	M + H		i	C24H37NO7
5	76	Ppyr_hemolymph_extract_469.291137695312_8.0686129		adduct of unknown 3	M + NH4		i	C24H37NO7
6	77	Ppyr_hemolymph_extract_507.222839355468_13.718764		unknown 4	M + H		iv	C26H34O10
7	77	Ppyr_hemolymph_extract_524.249481201171_13.718764		adduct of unknown 4	M + NH4		iv	C26H34O10
8	77	Ppyr_hemolymph_extract_549.233520507812_15.795523		unknown 5	M + H		iv	C28H36O11
9	78	Ppyr_hemolymph_extract_1114.52197265625_19.262445		unknown 6	—		ii	—
10	79	Ppyr_hemolymph_extract_531.222991943359_16.840886		unknown 7	M + H		iv	C28H34O10
11	80	Ppyr_hemolymph_extract_1082.49517822265_19.965233		aggregate ion of an unknown diacetylated lucibufagin isomer	2 M + NH4		iii	C28H36O10
12	80	Ppyr_hemolymph_extract_1100.50646972656_18.35508		unknown 8			iii	
13	80	Ppyr_hemolymph_extract_1082.49487304687_21.307386		aggregate ion of an unknown diacetylated lucibufagin isomer	2 M + NH4		iii	C28H36O10
14	80	Ppyr_hemolymph_extract_1082.49530029296_18.991166		aggregate ion of an unknown diacetylated lucibufagin isomer	2 M + NH4		—	C28H36O10
15	81	Ppyr_hemolymph_extract_517.243591308593_15.954252		unknown 9	M + H		iv	C28H36O9
16	81	Ppyr_hemolymph_extract_535.254180908203_12.579138		unknown 10	M + H		iv	C28H38O10
17	82	Ppyr_hemolymph_extract_1082.49475097656_15.123002		aggregate ion of diacetylated lucibufagin isomer 1	2 M + NH4	—	iv	C28H36O10
18	82	Ppyr_hemolymph_extract_1110.52667236328_17.431269		aggregate ion of monoacetylated, monopropylated lucibufagin isomer 2	2 M + NH4	—	iv	C29H38O10
19	82	Ppyr_hemolymph_extract_491.227569580078_12.962671	monoacetylated lucibufagin isomer 4		M + H	yes	iv	C26H34O9
20	82	Ppyr_hemolymph_extract_491.227661132812_10.204906	nonoacetylated lucibufagin isomer 1		M + H	yes	iv	C26H34O9
21	82	Ppyr_hemolymph_extract_533.237884521484_15.123002	diacetylated lucibufagin isomer 1		M + H	yes	iv	C28H36O10
22	82	Ppyr_hemolymph_extract_547.254028320312_17.431269	monoacetylated, mono propylated lucibufagin isomer 2		M + H	yes	iv	C29H38O10
23	82	Ppyr_hemolymph_extract_550.264221191406_15.123002		adduct of diacetylated lucibufagin isomer 1	M + NH4	—	iv	C28H36O10
24	82	Ppyr_hemolymph_extract_561.269470214843_19.784291	dipropylated lucibufagin isomer 3		M + H	no	iv	C30H40O10
25	82	Ppyr_hemolymph_extract_561.26953125_19.535629	dipropylated lucibufagin isomer 2		M + H	yes	iv	C30H40O10
26	82	Ppyr_hemolymph_extract_564.280517578125_17.431269		adduct of monoacetylated, mono propylated lucibufagin isomer 2	M + NH4	—	iv	C29H38O10
27	82	Ppyr_hemolymph_extract_574.264343261718_15.123002		adduct of diacetylated lucibufagin isomer 1	M + ACN + H	—	iv	C28H36O10
28	82	Ppyr_hemolymph_extract_578.296081542968_19.784291		adduct of dipropylated lucibufagin isomer 3	M + NH4	—	iv	C30H40O10
29	83	Ppyr_hemolymph_extract_1065.4677734375_15.366083		aggregate ion of diacetylated lucibufagin isomer 2	2 M + H	—	v	C28H36O10
30	83	Ppyr_hemolymph_extract_1082.49499511718_15.366083		aggregate ion of diacetylated lucibufagin isomer 2	2 M + NH4	—	v	C28H36O10
31	83	Ppyr_hemolymph_extract_491.227600097656_13.224755	monoacetylated lucibufagin isomer 5		M + H	no	iv	C26H34O9
32	83	Ppyr_hemolymph_extract_491.228057861328_11.943648	monoacetylated lucibufagin isomer 3		M + H	no	iv	C26H34O9
33	83	Ppyr_hemolymph_extract_533.238098144531_15.366083	diacetylated lucibufagin isomer 2		M + H	yes	iv	C28H36O10
34	83	Ppyr_hemolymph_extract_547.253814697265_17.719947	monoacetylated, mono propylated lucibufagin isomer 3		M + H	yes	iv	C29H38O10
35	83	Ppyr_hemolymph_extract_547.254211425781_17.046113	monoacetylated, mono propylated lucibufagin isomer 1		M + H	no	iv	C29H38O10
36	83	Ppyr_hemolymph_extract_550.264404296875_15.366083		adduct of diacetylated lucibufagin isomer 2	M + NH4	—	iv	C28H36O10
37	83	Ppyr_hemolymph_extract_561.269348144531_18.878158	dipropylated lucibufagin isomer 1		M + H	no	iv	C30H40O10
38	83	Ppyr_hemolymph_extract_561.269592285156_20.078779		unknown dipropylated lucibufagin isomer	M + H	—	iv	C30H40O10
39	83	Ppyr_hemolymph_extract_564.280212402343_17.719947		adduct of monoacetylated, monopropylated lucibufagin isomer 3	M + NH4	—	iv	C29H38O10
40	83	Ppyr_hemolymph_extract_578.295959472656_18.878158		adduct of dipropylated lucibufagin isomer 1	M + NH4	—	iv	C30H40O10
41	108	Ppyr_hemolymph_extract_449.217010498046_9.3229572	core lucibufagin isomer 2		M + H	yes	iv	C24H32O8
42	108	Ppyr_hemolymph_extract_491.227844238281_14.49087	monoacetylated lucibufagin isomer 6		M + H	no	iv	C26H34O9
43	109	Ppyr_hemolymph_extract_1615.72888183593_15.123002		aggregate ion of an unknown diacetylated lucibufagin isomer	3 M + NH4		—	C28H36O10
44	110	Ppyr_hemolymph_extract_449.217071533203_10.789573	core lucibufagin isomer 1		M + H	yes	iv	C24H32O8

^a^Feature number is arbitrary and correlates to order in Fig. 1 tree.

^b^Adducts identified for compounds with 2 or more co-eluting ions and accurate mass. Single ions are not identified to an adduct type.

^c^Fallon *et al*. manually removed adducts from MS2 similarity search and are represented by a dash.

^d^Dashes represent features that had no aligned MS/MS spectra by MZmine2.

^e^Proposed formula based on accurate mass measurements. All formulas are within 2 ppm of the experimental measurement.

### Mass spectral investigation of the unknowns

The additional 14 compounds include 10 lucibufagins of uncharacterized structure and 4 with precursor ions represented by the isomers identified by Fallon, *et al*.^10^. Unknown 1, 2 and 3 (of clusters 75 and 76) are possible new lucibufagins containing a nitrogen atom based on the single proposed molecular formula proposed within 2 ppm for each of these compounds. Further inspection identified unknown lucibufagins which may represent varying degrees of saturation or conversion of ketone groups to hydroxys (or vice versa) by mass differences of 2 Daltons. Unknown 7 (M + H *m/z*531.2230) in cluster 79 and unknown 10 (M + H *m/z*535.2543) in cluster 81 and vary by ±2 Daltons from that of diacetylated lucibufagins (M + H *m/z*533.2385) and unknown 5 (M + H *m/z*549.2335) of cluster 77 is 2 Daltons higher than monoacetylated-monopropylated lucibufagins (M + H m/z 547.2541). Unknown 6 (m/z1114.5221) of cluster 78 and 8 (*m/z*1100.5061) of cluster 80 are postulated to be aggregate ions given size of the precursor ions comparative to the lucibufagins. Unknown 9 (m/z 517.2436, cluster 89) and 4 (m/z 507.2287, cluster 94) are M + H adducts of previously unidentified lucibufagins. There are 3 features proposed as aggregate ions of new diacetylated isomers eluting between 18.94 and 21.01 minutes in cluster 80. A new dipropylated isomer (m/z 561.2695) was also identified in cluster 83.

CSI:FingerID is a tool that predicts and ranks candidate structures of experimental MS/MS data. Structure prediction using the diacetylated lucibufagin 1 MS/MS (Feature 21, Table 2) elicited the structure with highest similarity as lucibufagin C with 71% similarity. However the next highest similarity was very close at 70% and was predicted to be 12-hydroxymoorastatin (Supplementary Figure S3). The best predicted structures for unknowns 7 (64% similarity), 9 (71% similarity) and 10 (68% similarity) closely resembled the 12-hydroxymoorastatin structure (Supplementary Figure S4). It is possible these features, in the absence of a lucibufagin-like structure with the corresponding molecular formula were matched best to moorastatin-like compounds. Unknown 4 (65% similarity) and 5 (60% similarity) were predicted to be polycyclic compounds containing high numbers of acetyl groups, characteristics that are common to lucibufagins. The best matches for unknown 1, 2 and 3 contained single nitrogen atoms and multiple hydroxyl groups, however all 3 had matches below 60%.

### Comparison of BioDendro to FBMN of GNPS

The clustering of lucibufagins using BioDendro was compared to the FBMN module within the GNPS infrastructure. Application of BioDendro here used the MZmine2 (v2.53) exported feature list and the MGF from ProteoWizard which encompassed the entire 1,251 MS/MS positive ion mode spectra and after alignment, 492 features had an associated MS/MS spectra. Alignment of features to spectra for use in FBMN occurs during analysis using MZmine2 and a.txt feature list and MGF file are exported containing aligned features only. MZmine2 exported a total of 402 MS/MS spectra. The 44 lucibufagin features interrogated with the BioDendro output were examined within the molecular networks of FBMN (Fig. 1b). 42 of the 44 features were aligned an MS/MS by MZmine2 and were located in 5 networks generated using a cosine score of 0.7. From the aligned spectra, there exists a visual similarity of the structure between molecular networking and hierarchically clustered tree. The 5 new lucibufagins of clusters 75 and 76 found by BioDendro share a single network and all of features of cluster 82 and 83, bar 2 features, are also networked. Notable differences are the additional 4 nodes (nodes that are without a feature number) that form part of network iv) (Fig. 1b), comparison of the MS/MS spectra that created edges to the new nodes exhibit similarity based on neutral losses, a comparison that is optionally carried out in separate analyses within BioDendro.

Mass spectral searching was enabled during analysis using all MS/MS libraries accessible through GNPS. To date, GNPS has access to over 2.4 million MS/MS reference spectra (when considering GC-MS and LC-MS) from 27 different libraries^3^. From the 402 networked spectra, 10 features were matched to a reference spectra which did not include lucibufagin compounds.

### Application run time

Based on running on a Windows 7 PC, hierarchically clustering 492 spectra with BioDendro took 50 seconds. Molecular networking using FBMN is completed using a web-based server and analysis time will be dependent on the server load at that time. Several iterations of this analysis using identical settings at various times of the day took from 5 to 15 minutes to network 402 features.

## Discussion

Metabolomics studies often produce several hundred to several thousand unique MS/MS spectra for which metabolite identification or classification can be facilitated. Here, a protocol is presented that hierarchically clusters MS/MS spectra from metabolomics data and outputs fragment ion tables in an easy to interrogate manner. Many studies have explored numerous ways in clustering MS/MS spectra for proteomics analysis and mass spectral dereplication^16,28–31^ however, clustering MS/MS data for metabolomics has considerations not generally applicable to proteomics data such as distinguishing isobaric ions that are often dereplicated in many of these analysis pipelines^4^. The BioDendro protocol does not approach dereplication in the traditional manner of combining MS/MS data of same precursor mass and high similarity spectra regardless of retention time but rather a feature has a single alignment to an MS/MS spectrum within an m/z and retention time tolerance. In this way, all isobaric ions are represented individually.

Hierarchically clustering the MS/MS spectra of *P. pyralis* hemolymph facilitated the putative identification of 44 features containing a fragmentation pattern similar to that of the lucibufagins^25,26^. Using the tree to visualize clustering offers the opportunity to make easy, intuitive decisions regarding the structure of the clustered data. After review of the tree structure surrounding the analytes of interest it was particularly useful to adjust the distance threshold to adequately cluster the lucibufagins together.

Additionally, the ion tables and histograms output by BioDendro make it particularly easy to see the contribution of individual ions within the entirety of a single cluster, not presently available within FBMN. Identifying the ions which are heavily represented within a cluster can help to identify compounds which are not currently in mass spectral databases. Searching mass spectral databases^18–20,23,24^ for lucibufagins returned no hits, however MS/MS spectra for several lucibufagins were found in the literature^25–27^. Many natural product mass spectra exist in individual publications that haven’t been submitted to databases, especially those that were collected before the formation of mass spectral databases. Identification of those fragments which typify molecular structures could be used as search terms within literature to further leverage metabolite classification. Many natural product mass spectra exist in individual publications that haven’t been submitted to databases, especially those that were collected before the formation of mass spectra databases. Identification of those fragments which typify molecular structures could be used as search terms within literature to further leverage metabolite classification.

Comparison of the tree created in BioDendro against the molecular networking of FBMN exhibited clustering that was similar across both platforms. GNPS and the newer module FBMN have become gold standards for clustering metabolomics MS/MS data. However, difficulties in interrogating complete unknown spectral patterns led us to develop an alternative pipeline that allowed simple application with easily interrogable clusters and inspection of the spectral information behind those clusters. FBMN is a web-based application requiring a) upload of data to a webserver, b) data inputs produced by a limited number of data processing software c) production of the network and limited visualization within FBMN and d) further visualization in Cytoscape, an additional platform external to GNPS. Whereas hierarchical clustering in BioDendro a) can be run locally, b) accepts a feature list from any processing software, and c) produces simplified visualisation outputs not dependent on external software. Additionally, processing time for hierarchically clustering and outputting results is 7 times quicker than FBMN meaning optimization or modification of analysis parameters can be done with minimal downtime.

## Conclusion

Clustering MS/MS spectra for metabolomics data is often a way in which a user can interrogate feature classification or identification without the presence of authentic standards. It is a non-trivial undertaking that can often require a degree of technical knowledge regarding mass spectrometry and biological knowledge about the sample origin. Hierarchically clustering MS/MS spectra, visualized as a tree, presented an easily interrogable format, which coupled with cluster ion tables allowed users to make informed decisions for the classification of 29 unique compounds as lucibufagins. Accessing the ions which were highly represented within certain clusters improved the users’ confidence and ability in assigning a metabolite class to compounds that are not present in current MS/MS databases.

### Software availability statement

The BioDendro software developed in this study is available via https://github.com/ccdmb/BioDendro complete with example datasets and Jupyter Notebooks.

## Supplementary information

Supplementary Information.

Dataset 1.

## Data Availability

The Firefly data is freely available MetaboLights – project number MTBLS698.
