# Supplementary material for: Hierarchical clustering of MS/MS spectra from the firefly metabolome identifies new lucibufagin compounds
Source: Sci Rep. 2020 Apr 8;10:6043. doi: 10.1038/s41598-020-63036-1 (PMC7142086; doi:10.1038/s41598-020-63036-1)
Supplement: Supplementary file 1 — Supplementary Information. [file 41598_2020_63036_MOESM1_ESM.pdf]

Hierarchical clustering of MS/MS spectra from the firefly metabolome identifies new lucibufagin compounds

Catherine Rawlinson[1], Darcy Jones[1], Suman Rakshit[2], Shiv Meka[3], and Caroline S. Moffat[1], Paula Moolhuijzen[1].

[1] Centre for Crop and Disease Management, School of Molecular and Life Sciences, Curtin University, Bentley, Western Australia, Australia
[2] Statistics for the Australian Grains Industry-West, School of Molecular and Life Sciences, Curtin University, Bentley, Western Australia, Australia
[3] Curtin Institute for Computation, Curtin University, Bentley, Western Australia, Australia

Corresponding authors email: paula.moolhuijzen@curtin.edu.au (PM), catherine.rawlinson@postgrad.curtin.edu.au (CR)

Supplementary Information SI1

BioDendro requirements and parameters

The below settings are customisable for each use of BioDendro pipeline and available in the `help(BioDendro.pipeline)` cell of the BioDendro notebook. An expanded explanation is available here:

Minimum information required to run BioDendro pipeline

```
MGF_filename_here.mgf       Name of .mgf file
Component_filename_here.txt Name of .txt file
example: tree = BioDendro.pipeline("MSMS.mgf", "component_list.txt")
```

BioDendro pipeline will run with default parameters unless otherwise given. All below parameters in the following format:

```
parameter     description
              default value
              user options
```

```
mz_tol        m/z tolerance window to align MSMS spectra to component
                 list
              0.002
              Unlimited, dependent on instrument resolution
```

Using "mz_tol=" function allows users to define the m/z *window size to align the component list to the MS/MS pepmass in the MGF file. Value should reflect instrument resolution*

```
retention_tol retention time tolerance window to align MSMS spectra to
                 component list
              5
              Unlimited, recommended at half peak width
```

Using "retention_tol=" function allows users to define the retention time window to align an MS/MS spectrum to a component after searching for matching pepmasses. If multiple hits exist, BioDendro will use the closest retention time.

```
bin_threshold maximum difference between two consecutive masses before a
                 new mass bin is created
              0.0008
              Typical range 0.00001-1 Dalton
```

Using "bin_threshold=" function you can alter the m/z *difference between two consecutive masses that creates a new bin. All* m/z *fragments for all aligned spectra are listed and sorted based on mass. Fragments are placed in separate bins if their difference exceeds the threshold. Bin labels are presented as m/z average_m/z min_m/z max.* m/z *values are binned in BioDendro to allow comparison of fragments between MS/MS spectra. A bin threshold of 0.0008 was determined as appropriate for a data set collected at 70,000 resolution.*

```
scaling       Highest m/z within an MSMS spectrum is normalised to 1 and
                 all other masses are scaled to it
              False
              True or False
```

Using "scaling=" function will set whether you wish to filter out ions based on raw intensity or on scaled value. Each MS/MS spectra is scaled to its own highest intensity ion. If filtering is set to False, then this function serves no purpose

```
filtering     Ions within an MSMS spectra are removed based on intensity
                 set by 'eps' parameter
              False
              True or False
```

Using "filtering=" function allows the user to determine if ions are filtered out of the analysis before the resemblance matrix is built. Users can filter based on raw intensity or scaled values by combination with "scaling=" function.

```
eps           Scaled value for which ions below are removed
              None
              0.0 - 1.0 with scaling, otherwise data dependent without scaling
```

Using "eps=" function will set the level for which to remove ions below the set value. If "scaling=" is set to True, eps should be between 0 and 1, with 1 representing the highest intensity value. If scaling is set to False then enter a raw intensity value suitable for your data.

```
neutral       convert MSMS spectra to neutral loss spectra
              False
              True or False
```

Using the "neutral=" function, each m/z *within a spectra will be subtracted from the pepmass and the resulting spectrum will represent the mass lost during fragmentation.*

Supplementary Information SI1 continued

```
clustering method  Distance metric used for clustering
                   "jaccard"
                   "jaccard" or "braycurtis"
```

Jaccard similarity:

$$S_J = \frac{a}{a+b+c}$$

S_J= Jaccard index
a= number of ions common to both MSMS spectra
b= number of ions solely in the first spectrum
c= number of ions solely in the second spectrum

Bray-Curtin similarity:

$$S_{BC} = \frac{2a}{2a+b+c}$$

S_{BC}= Bray-Curtis index
a= number of ions common to both MSMS spectra
b= number of ions solely in the first spectrum
c= number of ions solely in the second spectrum

Jaccard and Bray-Curtis resemblance measures are interpretable as the proportion of ions shared. Bray-Curtis doubles the weighting of joint presence ions, whereas Jaccard has equal weighting on joint presence and single presence only ions. Bray-Curtis may be more applicable to data where MS/MS spectra contain many fragment ions (ie at higher collision energies).

```
cutoff             the y-axis value/distance to cut the dendogram and
                      form clusters
                   0.6
                   From 0 to 1
```

Using "cutoff=" function, the height of the cutoff for the dendrogram is altered. This can tighten or loosen the degree of similarity for of components within a cluster. We recommend using the default value as the pipeline allows users to update this value during data interrogation

```
width              width of dendogram output in pixels
                   900
                   Recommended maximum 1200
```

Using "width=" function allows you to alter the dimension of your dendrogram output. This value should reflect your monitor or workspace size for ease of viewing.

```
height             height of dendogram output in pixels. Branch labels are
                      included in this dimension
                   800
                   Recommended maximum 1200
```

Using "height=" function is similar to the "width" function, combination of these values allows you to set your dendrogram output size. Branch labels are included in this value, the longer your component identifiers are, the less space will be given to your dendrogram.

```
results_dir        directory to write per-cluster plots and tables to.
                   None (Will use `results_<datetime>` where
                      <datetime> is the current date and time in
                      YYYYMMDDHHmmSS format).
                   can be user defined
```

Using "results_dir=" function, the user can define a directory to store their data in. Default will generate a directory in the BioDendro folder.

```
out_html           file to write interactive dendogram plot to.
                   None (`results_dir\\simple_dendrogram.html`)
                   can be user defined
```

Using "out_html=" function allows the user to define a different directory for the dendrogram object. We recommend leaving as default and output to the results directory.

```
quiet              suppress pipeline messages
                   False
                   True or False
```

Using "quiet=" function will remove the notification in the pipeline during analysis.

Supplementary Information SI2

A. Elements used for molecular formula prediction of ions in table 1.
B. Additional elemental prediction parameters
C. Expanded version of table 1 (within manuscript) with the presented and next closest predicted elemental formula based on ppm error for ions highly represented in cluster 82 and 83

A

Elements in use				
Isotope	Min	Max	Double bond equivalents	Masss
16 O	0	15	0	15.995
12 C	0	30	1	12
1 H	0	60	-0.5	1.008

B

Charge	+1
Ring double bonds	-1 to 100

C

Fragment ion (m/z average_m/z min_m/z max)	Predicted molecular formula	ppm error
135.0443_135.0434_135.0448	C8H7O2	1.8
	C5H5ON3	11.7
205.0863_205.0840_205.0872	C12H13O3	1.8
	C16H13	-72.5
413.1965_413.1950_413.1970	C24H29O6	1.5
	C17 H33 O11	-12.7
105.0701_105.0698_105.0707	C8H9	2.1
	C4H9O3	147.3
121.0648_121.0644_121.0653	C8H9O	0.1
	C4 H9 O4	126.1
147.0805_147.0756_147.0812	C10H11O	1.1
	C6 H11 O4	104.1
185.0961_185.0924_185.0975	C13H13O	-1
	C9 H13 O4	82.5
151.0392_151.0388_151.0396	C8H7O3	1.5
	C12 H7	-99.5
265.1592_265.1540_265.1670	C19H21O	1.9
	C12 H25 O6	-20.2

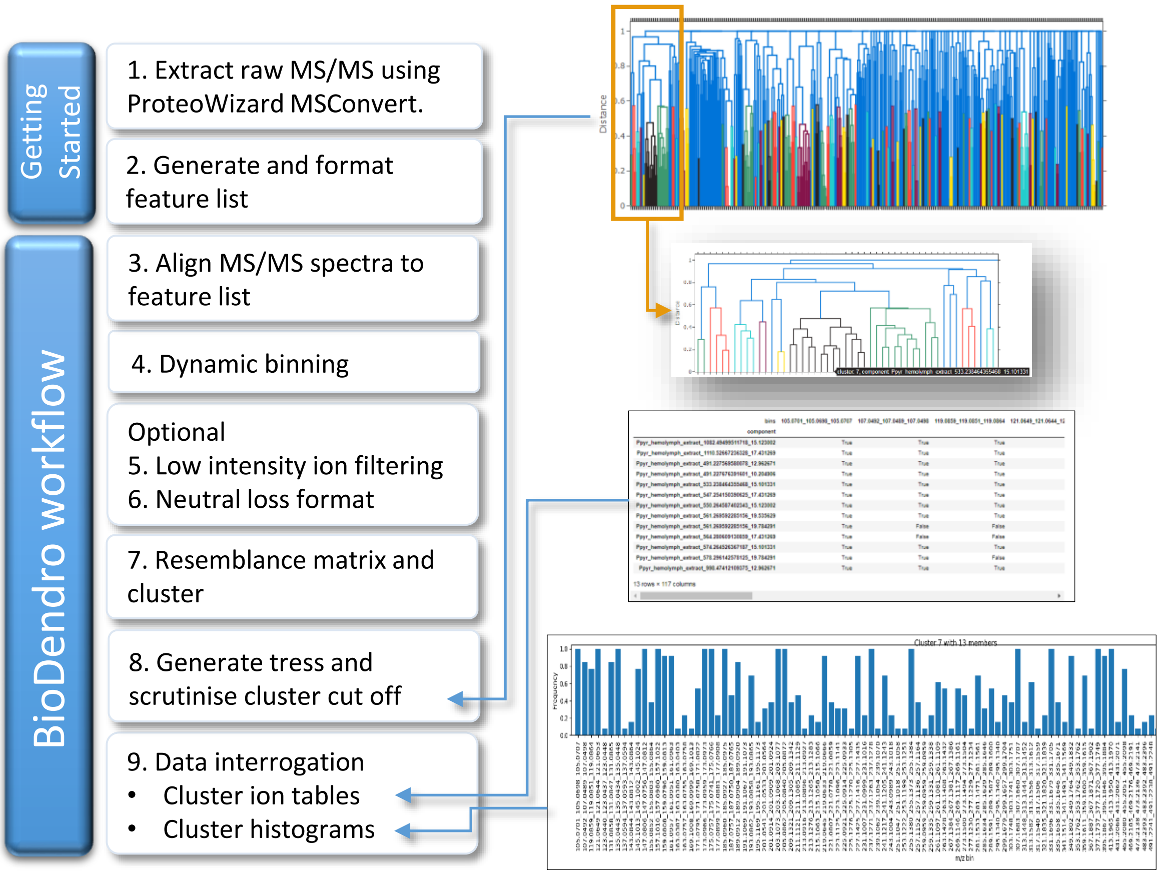

Supplementary Figure 1. BioDendro implementation and main functions are shown as a workflow and visualization results as plots. In the workflow 1) raw MGF formatted MS/MS file and 2) component lists are loaded into BioDendro, 3-7) with customizes workflow parameters and options for acquired data. Three outputs are generated to interrogate data structure, 8) interactive dendrogram, 9) cluster ion tables and cluster histograms.

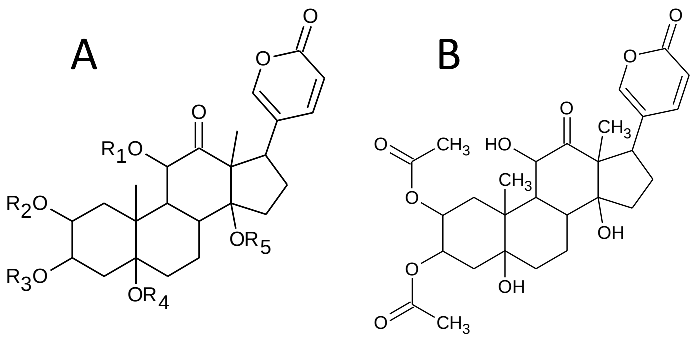

Lucibufagin backbone

Lucibufagin C
$C_{28}H_{36}O_{10}$

Supplementary Figure 2. Core molecular structure of the a) lucibufagin class of compounds and b) the diacetylated lucibufagin C.

**Mass detection**	
Noise MS1	5000
Noise MS2	1000
**ADAP Chromatogram Builder**	
Min group size in # of scans	8
Group intensity threshold	1000
Minimum height threshold	10000
m/z tolerance	0.001 Da or 5 ppm
**Smooth**	
Filter width	11
**Chromatogram Deconvolution**	
Algorithm	Local Minimum Search
Chromatogram threshold	5%
Search minimum retention time	0.1 min
Minimum relative height	1%
Minimum absolute height	30000
Minimum ratio of peak to edge	1.1
Peak duration range	0-1min
m/z center calculation	median
m/z range for MS2 scan pairing (Da)	0.005
RT range for MS2 scan pairing (min)	0.1
**Isotope Grouper**	
m/z tolerance	0.001 Da or 5 ppm
RT tolerance	0.2
Monotonic shape	yes
Maximum charge	2
Representative isotope	most intense
**Export/Submit to GNPS-FBMN**	
Merge MS/MS	no
Filter rows	only with MS2

Supplementary Table 1. Settings used within MZmine2 (v2.53), replicated from Fallon et al to produce the feature list used both for BioDendro and FBMN.

Parameter Options		Description	Parameter setting	
FBMN	BioDendro		FBMN	BioDendro
Filter precursor ion window	N/A	Deletes all ions within a +/- 17 Da window of precursor	off	N/A
Filter peaks in 50Da window	N/A	Only the 6 most intense fragment ions are retained per 50 Da window.	off	N/A
(from MZmine2)	Retention time tolerance (s)	RT tolerance for which an MS/MS spectra may be aligned to a feature	0.1 min (6 secs)	6 secs
(from MZmine2)	m/z tolerance (Da)	m/z tolerance for which an MS/MS spectra may be aligned to a feature	0.005	0.005
Fragment Ion Mass Tolerance	(see Bin threshold)	mass tolerance MS/MS spectral library matching and molecular networking	0.005	-
(see fragment ion mass tolerance)	Bin threshold	Maximum difference between two consecutive MS/MS product ions before a new bin is created	-	0.005
N/A	filtering	True/False - remove low intensity ions	N/A	TRUE
N/A	scaling	True/False - scale ions before filtering	N/A	FALSE
Minimum fragment ion intensity	eps	ions below this intensity will be deleted	5000	5000
N/A	Clustering method	Generates a pairwise similarity score between two MS/MS spectra	N/A	Bray-Curtis
N/A	Neutral Loss	Converts MS/MS spectra to neutral loss format for clustering	N/A	FALSE
Network/Clustering settings				
Minimum pairs cosine	(see cut-off)	Minimum cosine score to form an edge in a molecular network	0.7	N/A
(see minimum pairs cosine)	cut-off	Tree height where cut to generate clusters	N/A	0.6
Node TopK	N/A	Maximum number of nodes a single node may have.	10	N/A
Minimum matched fragment ions	N/A	Number of matched ions between to MS/MS spectra in order to form an edge	6	N/A
Maximum connected component size	N/A	Maximum size of molecular network	100	N/A

Supplementary Table S2. Parameter options and settings used for analysis

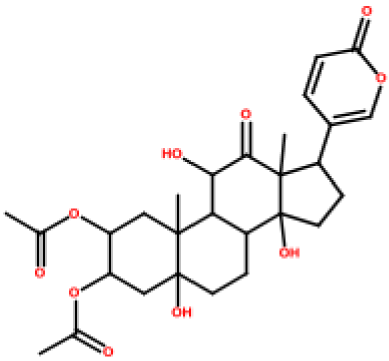

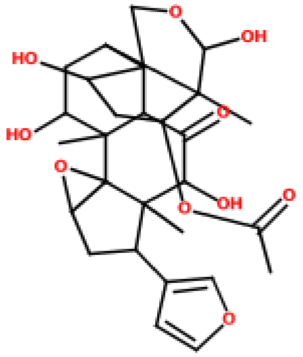

Diacetylated lucibufagin isomer 1
71% similarity

12-hydroxymoorastatin
70% similarity

Supplementary figure S3. The top 2 suggested structures by CSI:FingerID for diacetylated lucibufagin isomer 1 (feature 21 in table 2).

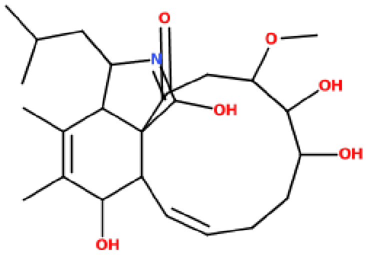

Unknown 1
55% similarity

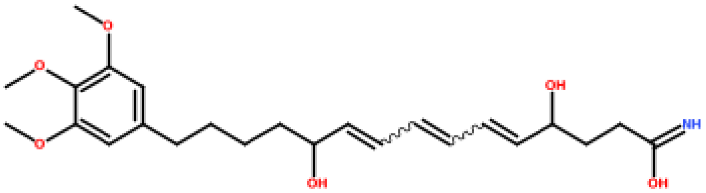

Unknown 2
46% similarity

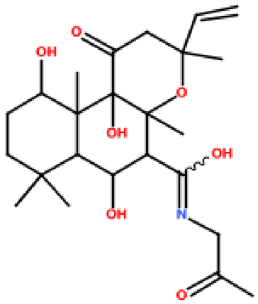

Unknown 3
56% similarity

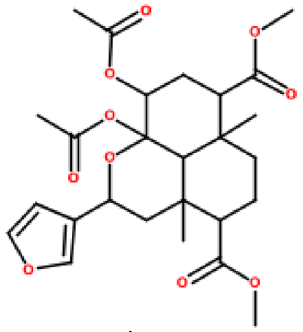

Unknown 4
65% similarity

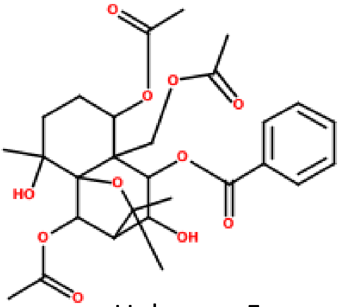

Unknown 5
60% similarity

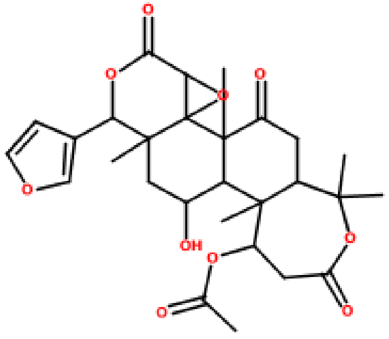

Unknown 7
64% similarity

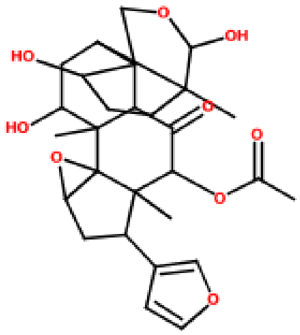

Unknown 9
71% similarity

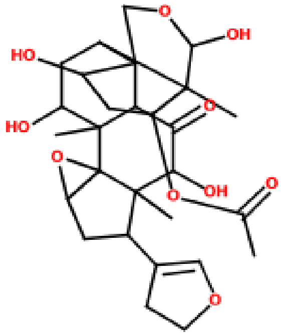

Unknown 10
68% similarity

Supplementary figure 4. Structures suggested by CSI:FingerID for unknown compounds (with an M+H adduct) within the lucibufagin clusters. The highest similarity of predicted to experimental MS/MS with the molecular formula predicted in table 2 is presented. The searched databases are Biocyc, CHEBI, GNPS, KEGG, KNApSAcK, Natural Products, Plantcyc, Pubchem, PubMed and YMDB.
